# Pathogen-Specific Bactericidal Method Mediated by Conjugative Delivery of CRISPR-Cas13a Targeting Bacterial Endogenous Transcripts

**DOI:** 10.1128/spectrum.01300-22

**Published:** 2022-08-11

**Authors:** Zihao Song, Yue Yu, Xinpeng Bai, Yiguo Jia, Jiayi Tian, Kui Gu, Mengyu Zhao, Changyu Zhou, Xiangyu Zhang, Hongning Wang, Yizhi Tang

**Affiliations:** a Wuyuzhang Honors College, Sichuan University, Chengdu, Sichuan, China; b College of Life Sciences, Sichuan University, Chengdu, Sichuan, China; c Key Laboratory of Bio-Resource and Eco-Environment of Ministry of Education, Sichuan University, Chengdu, Sichuan, China; d Animal Disease Prevention and Food Safety Key Laboratory of Sichuan Province, Chengdu, Sichuan, China; Health Canada

**Keywords:** CRISPR-Cas13a, conjugative delivery, precise bactericidal methods, CRISPR, *Salmonella* Typhimurium

## Abstract

The emergence of antibiotic-resistant bacteria threatens public health, and the use of broad-spectrum antibiotics often leads to unintended consequences, including disturbing the beneficial gut microbiota and resulting in secondary diseases. Therefore, developing a novel strategy that specifically kills pathogens without affecting the residential microbiota is desirable and urgently needed. Here, we report the development of a precise bactericidal system by taking advantage of CRISPR-Cas13a targeting endogenous transcripts of Salmonella enterica serovar Typhimurium delivered through a conjugative vehicle. *In vitro*, the CRISPR-Cas13a system exhibited specific killing, growth inhibition, and clearance of *S.* Typhimurium in mixed microbial flora. In a mouse infection model, the CRISPR-Cas13a system, when delivered by a donor Escherichia coli strain, significantly reduced *S.* Typhimurium colonization in the intestinal tract. Overall, the results demonstrate the feasibility and efficacy of the designed CRISPR-Cas13a system in selective killing of pathogens and broaden the utility of conjugation-based delivery of bactericidal approaches.

**IMPORTANCE** Antibiotics with broad-spectrum activities are known to disturb both pathogens and beneficial gut microbiota and cause many undesired side effects, prompting increased interest in developing therapies that specifically eliminate pathogenic bacteria without damaging gut resident flora. To achieve this goal, we developed a strategy utilizing bacterial conjugation to deliver CRISPR-Cas13a programmed to specifically kill *S.* Typhimurium. This system produced pathogen-specific killing based on CRISPR RNA (crRNAs) targeting endogenous transcripts in pathogens and was shown to be effective in both *in vitro* and *in vivo* experiments. Additionally, the system can be readily delivered by conjugation and is adaptable for targeting different pathogens. With further optimization and improvement, the system has the potential to be used for biotherapy and microbial community modification.

## INTRODUCTION

Since the discovery of penicillin (1, 2), antibiotics have played an essential role in fighting against bacterial infections. Due to the overuse and stagnation in the discovery of new antibiotics (3, 4), drug-resistant bacteria have emerged rapidly and are recognized as a major public health threat around the world. Based on projections, social and public expenditures attributable to antibiotic-resistant pathogens will reach $100 billion by 2050 (5). To combat this trend, new antimicrobial therapies, such as antibiotic adjuvants (6) and antimicrobial peptides (7, 8), are needed and are being developed. Notably, antibiotics commonly used to fight pathogens also target commensal bacteria, disturbing the composition of gut microbiota and causing unintended consequences (diseases). Therefore, bacterial species-specific and precise antimicrobial technologies are desirable and are being developed (9).

CRISPR-Cas systems, widespread in bacteria and archaea, are responsible for adaptive cellular immunity against exogenous DNA (plasmids and phage) (10–15). The CRISPR-Cas9 system introduces double-strand breaks (DSBs) in target DNA sites guided by specific CRISPR RNAs (crRNAs) and a transactivating CRISPR RNA (tracrRNA), making it a powerful gene-editing tool (16, 17). Meanwhile, DSBs cause replication fork collapse and cell death, providing the CRISPR-Cas9 system with the potential for a precise antibacterial tool (18–25). CRISPR-Cas13a, a type VI CRISPR system targeting and cleaving single-stranded RNA (ssRNA), has both precise and promiscuous cutting activities after recognizing the target ssRNA (26–29), resulting in specific gene inactivation and bacterial growth inhibition to defend against phage infection without any damage to DNA (30). This property demonstrates a great antimicrobial potential (31). The CRISPR-Cas13a system has attomolar sensitivity (28), and its crRNA design is easier and does not require the NGG sequence of the PAM region for LwaCas13a (30). In addition, since it targets RNA rather than DNA, CRISPR-Cas13a can target genes carried by plasmids and kill those strains instead of just removing the plasmid like the DNA-targeted CRISPR system. It also spares some strains that do not express pathogenic genes due to mutations, leading to higher target selectivity. Previous studies have investigated the bactericidal effect of the Cas13a system for targeting plasmid-carried genes (31), but few studies have used this system to eliminate pathogens species specifically.

As a nucleic acid-based antibacterial, an effective and continuous delivery vehicle is necessary for CRISPR systems. Phages possess a strong infection activity for bacteria and are the most commonly used delivery vehicle of CRISPR systems to achieve gene-specific bacterial killing (18–22, 24, 25, 31). However, the narrow spectrum of phages limits their application. Plasmid-based conjugation is a viable alternative for CRISPR system delivery (23, 32, 33), having the advantages of long duration of action, accessible usability, ease of design, no specific receptor required, and suitability for different pathogens (34). Therefore, conjugative plasmids are suitable methods for conveying nucleic acid-based antibacterial methods. Recent studies have used conjugative plasmids in *trans* or in *cis* to deliver the CRISPR-Cas9 system for the purpose of removing drug-resistant plasmids (35, 36) or killing specific bacteria directly (32, 37), showing high efficacy and usability *in vitro* and *in vivo*. However, conjugative plasmids with CRISPR-Cas13a have not been used for the specific killing of bacterial pathogens.

**FIG 1:**
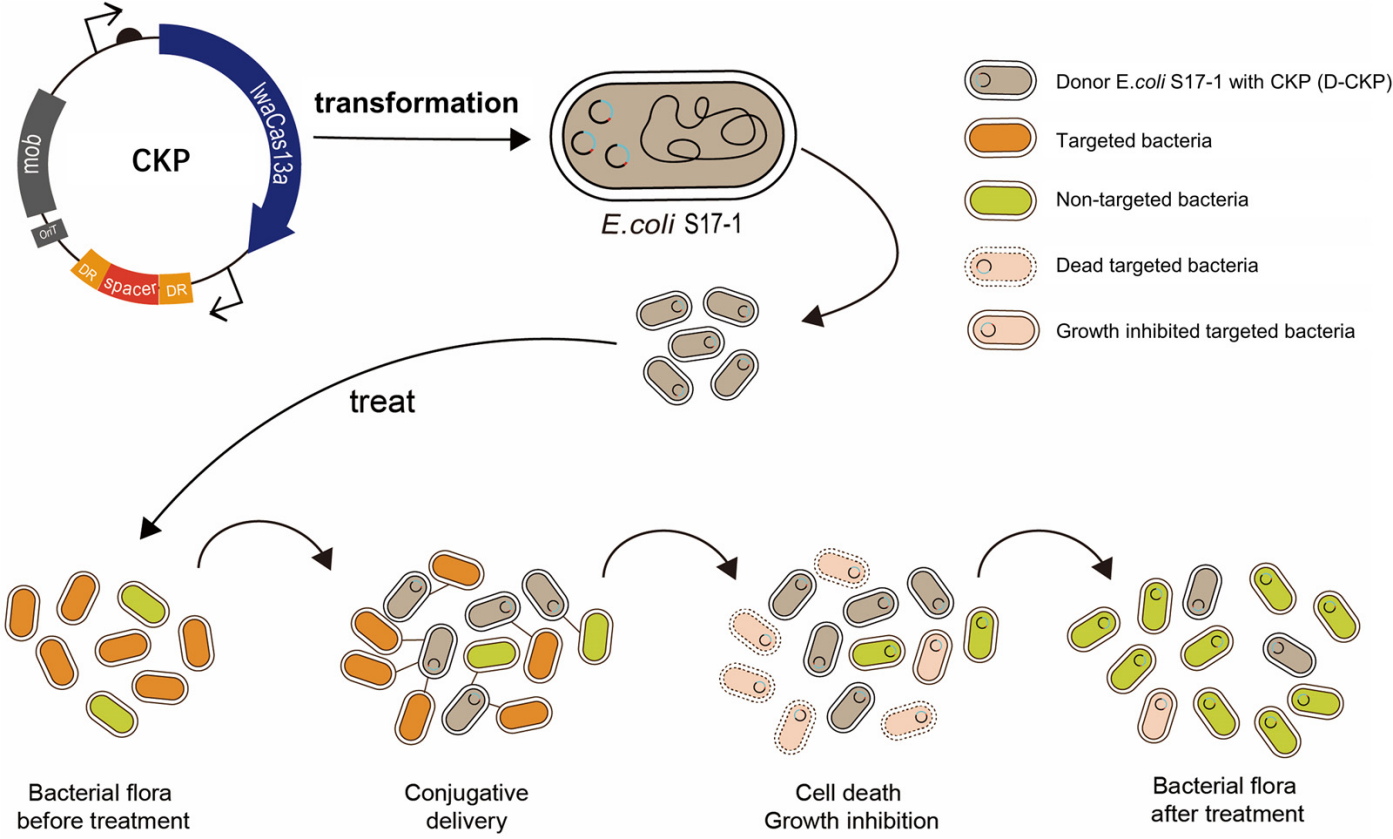
Schematic representation of CRISPR-Cas13a-mediated killing of target pathogens. Cas13a-based killing plasmids (CKPs) are transformed into E. coli S17-1, which serves as a donor and then delivers the CKPs to the target bacterial cells through transconjugation. When CKP proteins recognize specific bacteria, they will kill or cause growth inhibition of the target bacteria. This results in a decrease in the number of specific bacteria in a mixed flora.

In this work, we developed a strategy using the CRISPR-Cas13a system delivered by a *trans*-conjugative plasmid to specifically kill Salmonella enterica subspecies *enterica* serovar Typhimurium. The *trans*-conjugative delivery system is named the Cas13a-based killing plasmid (CKP), whose targets are endogenous transcripts of *S.* Typhimurium (Fig. 1). We detected the exact bactericidal effect of the CRISPR-Cas13a system and showed that this system is effective in killing Salmonella using both *in vitro* and *in vivo* experiments.

## RESULTS

### Construction of CKPs.

To construct Cas13a-based killing plasmids (CKPs), we used pBBR1-MCS2 (38) as the backbone (including *oriT*, relaxase gene *mob*, and kanamycin resistance gene) and inserted the chloramphenicol resistance gene (*CmR*), the crRNA transcription cassette, and the *LwaCas13a* gene (Leptotrichia wadei) (29) into the backbone. We designed 5 crRNAs targeting endogenous transcripts of *S.* Typhimurium, including 2 for the *S.* Typhimurium gene *dnaA* (CKP-*dnaA*1 and CKP-*dnaA*2, encoding chromosomal replication initiator protein), two for the gene *katG* (CKP-*katG*1 and CKP-*katG*2, encoding the catalase), one for the gene *hilA* (CKP-*hilA*, encoding transcriptional regulator HilA), and one crRNA for the nontarget (CKP-nontarget). Of these three genes, *dnaA* is essential for the growth of *S.* Typhimurium, while the other two are nonessential genes. Since *dnaA* and *katG* genes are also present in Escherichia coli, the sequence alignments of target sites between E. coli and *S.* Typhimurium are presented in Fig. S1b in the supplemental material. E. coli S17-1 (39), which carries the transfer (*tra*) regions of IncP-type plasmid RP4, served as the donor strain. In *in vitro* experiments, the pBAD promoter was used to control the expression of LwaCas13a; thus, it can be induced by l-arabinose and repressed by d-glucose with regulation of the *araC* gene assembled in CKPs (Fig. S1a, left). In *in vivo* experiments, we replaced pBAD in 4 CKPs (CKP-*dnaA*1, CKP-*katG*2, CKP-*hilA*, and CKP-nontarget) with the constitutive promoter pJ23104 to ensure the expression of the *LwaCas13a* gene, and these plasmids were named cCKPs (Fig. S1a, right). Next, we transferred CKPs and cCKPs into E. coli S17-1, which served as a conjugative donor, and these plasmid-containing E. coli donor strains were named d-CKPs and d-cCKPs, respectively.

### CKPs mediated the elimination of *S.* Typhimurium through conjugation *in vitro*.

**FIG 2:**
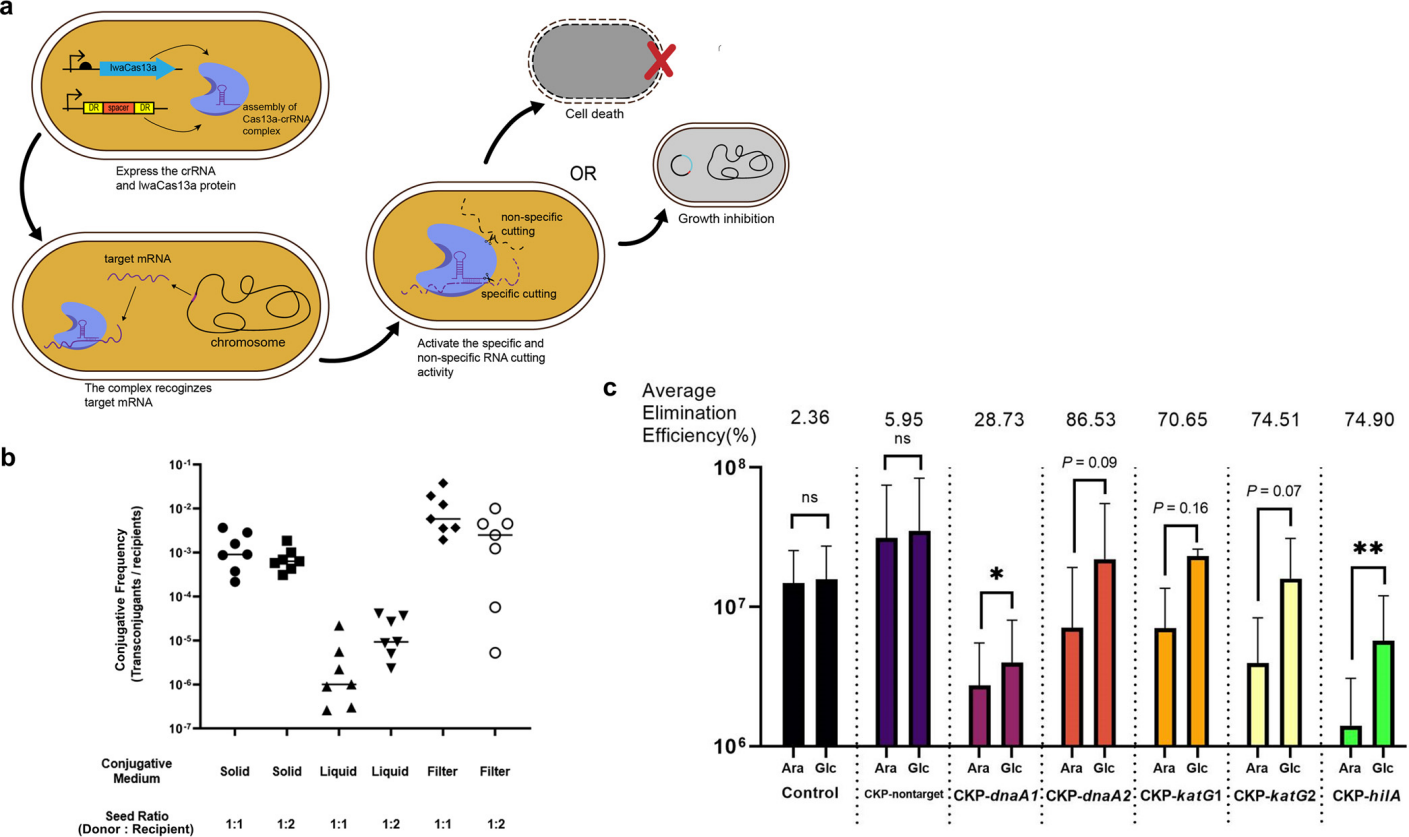
CKP-mediated elimination of *S.* Typhimurium by conjugation *in vitro*. (a) Diagrams showing the possible mechanisms of CRISPR-Cas13a-mediated elimination. CKP expresses the LwaCas13a protein and crRNA to form a CRISPR complex, which activates targeted and random ssRNA cleavage activity when the complex recognizes its targeted endogenous mRNA, causing death or growth inhibition of the target bacteria, thus exhibiting a removal effect. (b) Transfer efficiency of CKP from E. coli S17-1 to *S.* Typhimurium under different conjugative conditions (LB agar, LB broth, and 50-μm Millipore filter) and seed ratios (donor/recipient, 1:1 or 1:2). Horizontal bars represent the mean of data from at least 7 biological replicates. (c) Elimination efficiency after Cas13a induction. Bars represent the standard deviation of data from 3 biological replicates, and each dot represents a biological replicate. *P* values were determined by ratio-paired Student's *t* test (ns, not significant; *, *P* < 0.05; **, *P* < 0.01; ***, *P* < 0.001).

To select the optimal conjugation media and seed ratio, *S.* Typhimurium and d-CKP-nontarget (E. coli S17-1 with CKP-nontarget) were used as recipient and donor strains, respectively. A Millipore filter, LB broth, and LB agar were tested with a 1:1 or 1:2 seed ratio (donor/recipient) at 37°C for 20 h (Fig. 2b). Transconjugants and recipients were isolated on LB plates supplemented with ampicillin, kanamycin, and chloramphenicol or only with ampicillin. The results showed that the microporous filter had the highest efficiency of ~5 × 10^−2^, LB agar had a slightly lower efficiency of ~10^−3^, and LB broth had the lowest frequency of ~10^−6^. The seed ratio hardly affected the conjugation efficiency (Fig. 2b). Thus, in the following *in vitro* experiments, microporous filters were used as conjugative media in a 1:1 seed ratio.

We assessed the elimination efficacy of each CKP by comparing the ratio of *S.* Typhimurium transconjugant CFU under the conditions that LwaCas13a was induced by arabinose and repressed by glucose. In addition, CKP-free *S.* Typhimurium (control group) was used to assay the impact of arabinose and glucose. We found that the crRNA without targeting had little elimination (~2%) (Fig. 2c), indicating that CRISPR-Cas13a alone had no elimination activity for *S.* Typhimurium in the absence of the target sequence. Next, we evaluated the elimination of *S.* Typhimurium by inducing the CRISPR-Cas13a systems with targets. Two CKPs targeting the *dnaA* gene had elimination efficiencies of 28.7% and 86.5%, respectively. When targeting the *katG* gene and *hilA* genes, the elimination efficiencies were 74.9% (CKP-*katG*1), 70.7% (CKP-*katG*2), and 74.5% (CKP-*hilA*), respectively (Fig. 2c). These results indicate that CKPs can mediate the elimination of *S.* Typhimurium by designed crRNAs in an *in vitro* conjugation assay. Moreover, we cultured the plate with arabinose for 168 h, finding that no new small colonies appeared on those plates (Fig. S3). This suggested that this elimination effect might be due to bactericidal activity. As shown in Fig. S3, the reduction in colony diameters in CKP groups compared with CKP-nontarget or control group seemed to indicate an effect of the growth inhibition with a targeted CRISPR-Cas13a system.

Additionally, to test the specificity of CKPs, we replaced *S.* Typhimurium with E. coli Trelief 5α (with ampicillin resistance) as the recipient strain. The “control” group here was CKP-free E. coli. The results showed that none of the CKPs killed E. coli (Fig. S2). These results indicate that CKPs with crRNAs targeting *S.* Typhimurium have species-specific removal activity *in vitro* through conjugation transfer.

### Defining the killing effect of CRISPR-Cas13a against Salmonella when targeting endogenous transcripts.

The CRISPR-Cas13a system can inhibit the growth of bacteria through random cleavage activity, thus making phage infection fail (30). This growth inhibitory activity also leaves questions about whether the CRISPR-Cas13a system can be a bactericidal weapon. In previous experiments, we showed that CKP has elimination activity against *S.* Typhimurium, However, some groups, such as *dnaA*2 (*P = *0.09), *katG*1 (*P = *0.16), and *katG*2 (*P = *0.07), showed no statistically significant decrease under the induction of the Cas13a system (Fig. 2c), so further verification of the bactericidal effect of CRISPR-Cas13a is required. Here, we used a method for further assaying the killing effect of CRISPR-Cas13a when targeting endogenous transcripts (Fig. 1 and Fig. 2a). We precultured CKP-carrying *S.* Typhimurium in LB broth supplemented with d-glucose to an *A*_600_ of ~0.5, in which the expression of Cas13a was repressed. Then, the cultures were transferred to phosphate-buffered saline (PBS) supplemented with 0.2% l-arabinose or d-glucose at a ratio of 1:100 and incubated for 24 h. Samples were coated on “recovery plates,” which were supplemented with 0.2% glucose to repress Cas13a expression, and the difference in the number of CFU between the time of inoculation and after treatment was calculated to detect the killing efficiency.

**FIG 3:**
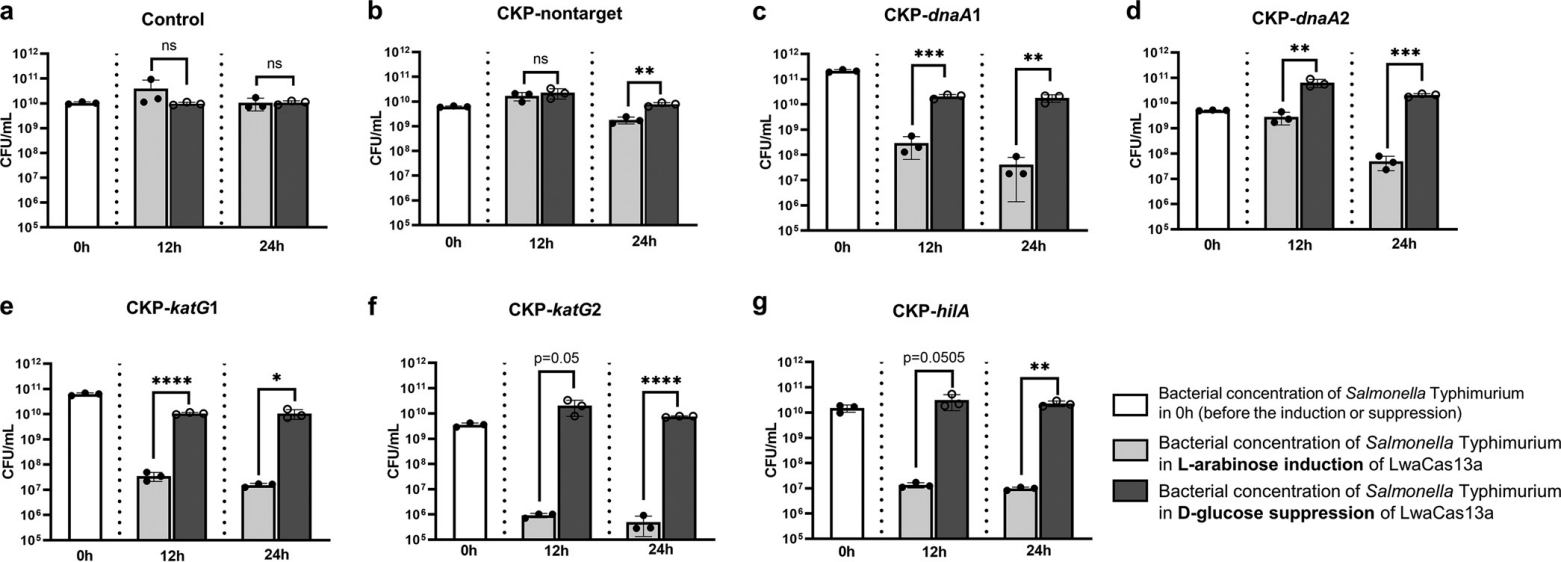
CRISPR-Cas13a-mediated bacterial death. *S.* Typhimurium counts as determined (*n* = 3 for each time point) at the indicated time points after induction with l-arabinose or repression with d-glucose. (a) *S.* Typhimurium without plasmid; (b) *S.* Typhimurium carrying CKP-nontarget; (c) *S.* Typhimurium carrying CKP-*dnaA*1; (d) *S.* Typhimurium carrying CKP-*dnaA*2; (e) *S.* Typhimurium carrying CKP-*katG*1; (f) *S.* Typhimurium carrying CKP-*katG*2; (g) *S.* Typhimurium carrying CKP-*hilA*. Bars represent the standard deviation of data from 3 biological replicates, and each dot represents data from a biological replicate. *P* values were determined by two-sided Student’s *t* test. (ns, *P > *0.05; *, *P < *0.05; **, *P* < 0.01; ***, *P* < 0.001; ****, *P < *0.001).

To examine the killing effect of CRISPR-Cas13a when targeting endogenous transcripts, *S.* Typhimurium without CKPs cultured in PBS was used as the control group, and we found that the number of bacterial CFU had no significant variability under the condition mentioned above over 24 h, and the addition of d-glucose and l-arabinose hardly affected the CFU (Fig. 3a). For the CKP-nontarget group, the group treated with arabinose decreased by approximately 0.5 orders of magnitude compared to the group treated with glucose at 24 h (1.80 × 10^9^ versus 7.67 × 10^9^ CFU/mL) (Fig. 3b), which indicated a slight metabolically toxic effect in the expression of Cas13a. However, for all five CKPs targeting endogenous transcripts, the *S.* Typhimurium density showed a significant decrease (Fig. 3c through g), with the Cas13a-induced groups having at least 2 orders of magnitude fewer viable cells than the Cas13a-inhibited groups 24 h after treatment. The CKP-*katG*2 group showed the highest killing efficiency, with a difference of 4 orders of magnitude (4.93 × 10^5^ versus 6.33 × 10^9^) (Fig. 3f). For the spot-plating experiments in Fig. S4, each small image represents 10 to 15 μL of bacterial solution in a certain dilution fold. In addition to arabinose, cell densities of CKP-*katG*1, CKP-*katG*2, and CKP-*hilA* groups were 10^6^ to 10^7^ CFU/mL, and CKP-*dnaA*1 and CKP-*dnaA*2 were approximately 10^7^ to 10^8^ CFU/mL, while groups treated with glucose showed 10^9^ to 10^10^ CFU/mL. Spot-plating images presented similar results to the corresponding bar charts. These data suggested that the CRISPR-Cas13a system targeting endogenous transcripts indeed caused the precise killing of target pathogens.

### CKP-mediated growth inhibition of *S.* Typhimurium.

**FIG 4:**
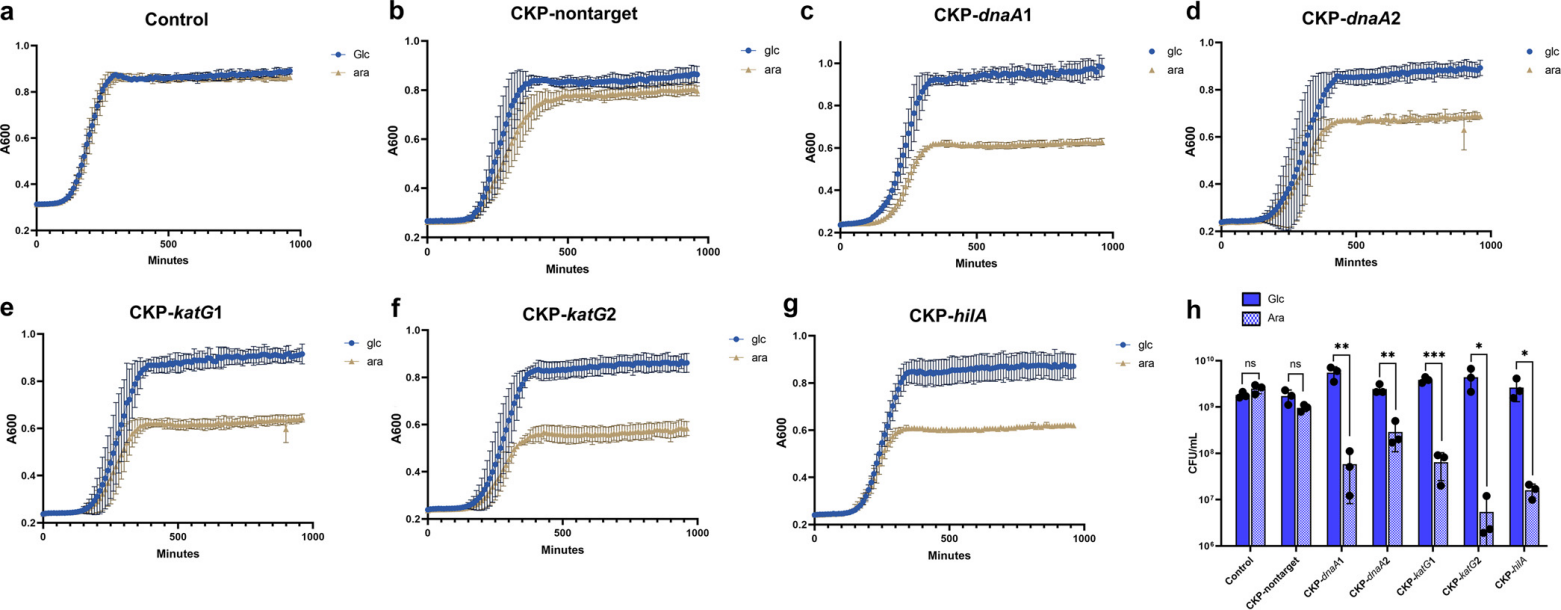
Growth curve analysis demonstrating CRISPR-Cas13a-mediated growth inhibition. (a to g) The *A*_600_ values of CKPs-carrying *S.* Typhimurium strains cultured in LB broth supplemented with d-glucose (blue) or l-arabinose (brown) were plotted every 10 min for 16 h. (h) The CFU number of *S*. Typhimurium after 16 h of culture supplemented with d-glucose or l-arabinose. Bars represent the standard deviation of data from 3 biological replicates, each dot represents data from a biological replicate, and *P* values were determined by two-sided Student’s *t* test (ns, *P > *0.05; *, *P < *0.05; **, *P < *0.01; ***, *P < *0.001; ****, *P < *0.001).

To further investigate whether the CRISPR-Cas13a system had a bacteriostatic activity (Fig. 1 and Fig. 2a) in addition to its bactericidal activity, individual CKP-carrying *S.* Typhimurium strains and *S.* Typhimurium without CKPs (control group) were cultured in LB broth supplemented with l-arabinose or d-glucose, and the absorbance at 600 nm (*A*_600_) was measured every 10 min for 960 min (16 h). In the control group of *S.* Typhimurium, the *A*_600_ values were basically the same in the two cultures treated with arabinose and glucose at 16 h (Fig. 4a), and there was no significant difference in the number of CFU (Fig. 4h), indicating that the addition of glucose or arabinose did not affect the growth of bacteria or have the same effect. For CKP-nontarget, the difference in *A*_600_ and CFU between the two treatments was approximately 0.05 and 0.8 orders of magnitude, respectively. However, the difference was not statistically significant (Fig. 4b). However, as shown in Fig. 4b and Fig. S5, a lagging growth effect occurred at 250 min, which might mean that the expression of Cas13a mediated a toxic effect on growth.

For the two CKPs targeting the genes *dnaA*1 and *dnaA*2, the differences in *A*_600_ between the cultures treated with arabinose and glucose after 16 h of incubation were 0.35 (CKP-*dnaA*1) (Fig. 4c; Fig. S5) and 0.205 (CKP-*dnaA*2) (Fig. 4d; Fig. S5), respectively. The cultures treated with arabinose exhibited an approximately 2-log and 1-log reduction in the numbers of CFU after 16 h of incubation for CKP-*dnaA*1 and CKP-*dnaA*2, respectively (*P = *0.0067 and *P = *0.0036) (Fig. 4h). Similarly, for the groups targeting two *katG* genes, the peak *A*_600_ values differed by 0.271 (CKP-*katG*1) (Fig. 4e; Fig. S5) and 0.278, respectively (CKP-*katG*2) (Fig. 4f; Fig. S5), and the cultures treated with arabinose exhibited an approximately 2-log and 3-log reduction in the numbers of CFU for CKP-*katG*1 and CKP-*katG*2, respectively (*P = *0.0003 and *P = *0.0297) (Fig. 4h). For CKP-*hilA*, the differences in the *A*_600_ value and CFU were approximately 0.251 and 2 log, respectively, showing a significant difference (*P = *0.0266). These data suggest that the CRISPR-Cas13a system does produce a growth-inhibitory effect upon recognition of the target RNA, and the combined effect of killing and growth inhibition of this system on the target bacteria makes it a promising species-specific elimination technology.

### CKP-mediated reduction of *S*. Typhimurium in mixed flora.

**FIG 5:**
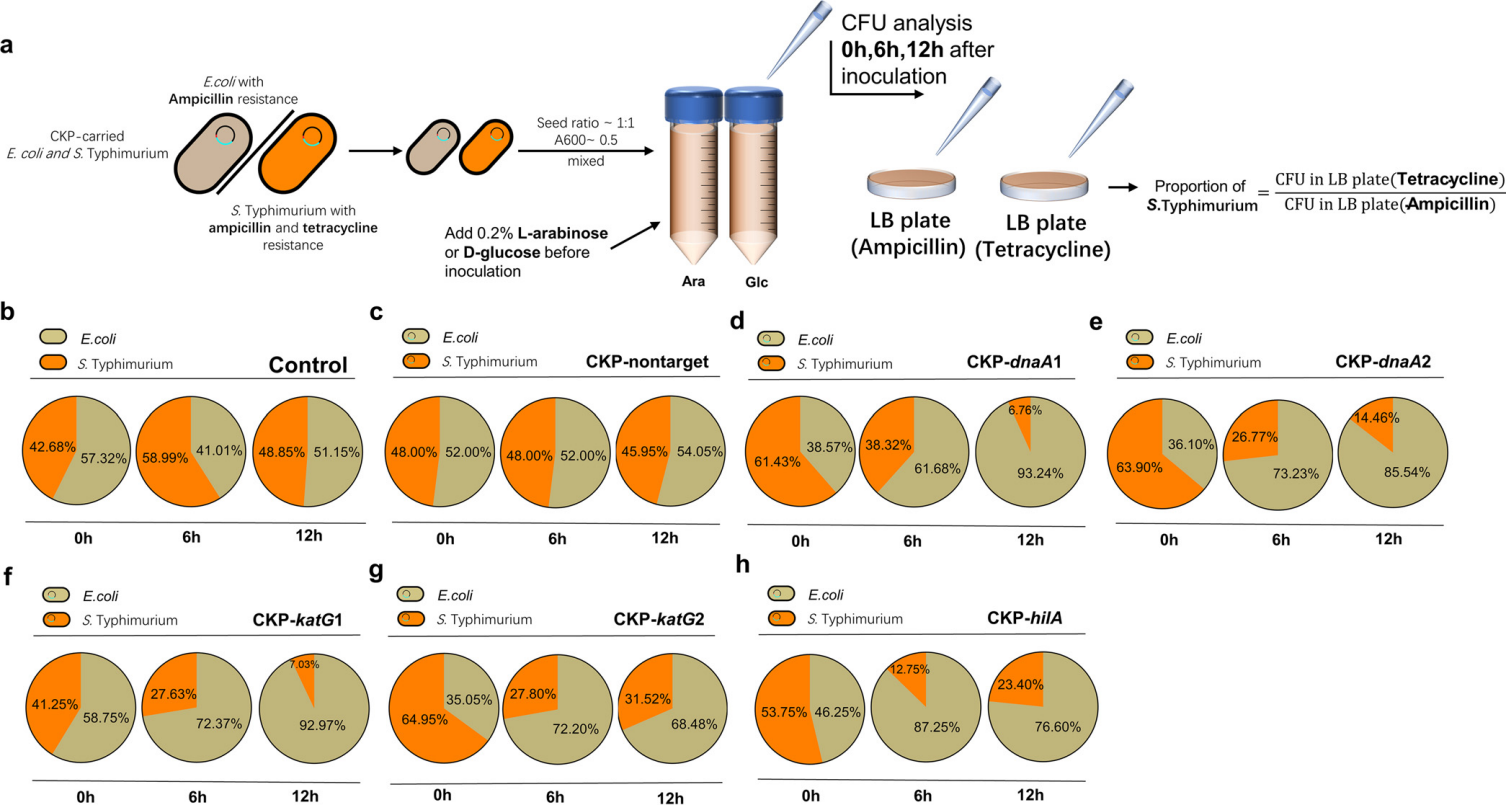
CKP-mediated reduction in the proportion of *S.* Typhimurium in mixed flora. (a) Schematic illustration of the mixed bacterial flora experiment. (b to h) Sector plots visualizing the percentage of E. coli and *S.* Typhimurium in flora, with each dot representing 1%. The orange color represents *S.* Typhimurium, and the gray-green color represents E. coli. All assays included 3 biological replicates. (b) *S.* Typhimurium without plasmid; (c) *S.* Typhimurium carrying CKP-nontarget; (d) *S.* Typhimurium carrying CKP-*dnaA*1; (e) *S.* Typhimurium carrying CKP-*dnaA*2; (f) *S.* Typhimurium carrying CKP-*katG*1; (g) *S.* Typhimurium carrying CKP-*katG*2; (h) *S.* Typhimurium carrying CKP-*hilA*.

To further verify the bactericidal and bacteriostatic effects of CKPs on the target bacteria in mixed flora (Fig. 1), we assayed the conjugative delivery frequency in two species of bacterial flora consisting of *S.* Typhimurium (with ampicillin and tetracycline resistance) and E. coli Trelief 5α (with ampicillin resistance) at 1:1 seed ratio into LB broth. We put d-CKP-nontarget into the flora as a conjugative donor and found that the conjugative frequency to E. coli was ~10% in 18 h and ~20.3 in 36 h of conjugation, which was significantly higher than that to *S.* Typhimurium (~0.5% in 18 h and ~1.1% in 36 h) (Fig. S6a). Due to the large difference in conjugation frequency between the two species, it is difficult to observe a reduction in *S.* Typhimurium population density in the flora of transconjugants after direct conjugation (Fig. S6b). We then mixed CKP-carrying E. coli and *S.* Typhimurium strains at a seed ratio of approximately 1:1, making the reduction in population density of *S.* Typhimurium in the flora more pronounced by inducing the expression of Cas13a. The control group means CKP-free *S.* Typhimurium and E. coli. Samples were enumerated at 0 h (the time of inoculation), 6 h, and 12 h (Fig. 5a). The CFU on the plate supplemented with ampicillin were considered the bacterial density of the entire flora, while the number of CFU on the plate supplemented with tetracycline was considered the population density of *S.* Typhimurium. From this differential plating, the change in the percentage of target bacteria in the mixed community after induction of the expression of the CRISPR-Cas13a system was calculated.

We used sector plots to visualize the percentage of E. coli and *S.* Typhimurium in flora. The orange color represents *S.* Typhimurium, and the gray-green color represents E. coli. The percentage of *S.* Typhimurium in the control group at initial inoculation changed from 42.68% initially to 58.99% after 6 h and 48.85% after 12 h of incubation with the addition of arabinose, indicating that the addition of arabinose did not affect the percentage of *S.* Typhimurium in flora. The percentage of CKP-nontarget-carrying *S.* Typhimurium was 48% at 0 h and 6 h and 45.95% at 12 h, indicating that in the absence of the target, the CRISPR-Cas13a system did not affect the percentage of *S.* Typhimurium in the flora either.

For the flora with CKP-*dnaA*1, the percentage of *S.* Typhimurium decreased from 61.43% initially to 38.32% at 6 h and then to 6.76% at 12 h after the addition of arabinose to induce Cas13a expression. For CKP-*dnaA*2, the percentage of *S.* Typhimurium also decreased from 63.90% initially to 26.77% at 6 h and 14.46% at 12 h. For the two CKP strains targeting the *katG* gene, their initial proportions of *S.* Typhimurium in the flora were 41.25% (CKP-*katG*1) and 64.95% (CKP-*katG*2), respectively, and then decreased to 27.63% (CKP-*katG*1) and 27.80% (CKP-*katG*2) at 6 h, while after 12 h of incubation in LB broth with arabinose, the *S.* Typhimurium percentages were 7.03% and 31.52%, respectively. The percentages of *S.* Typhimurium in the CKP-*hilA* group at 0 h, 6 h, and 12 h were 53.75%, 12.75%, and 23.40%, respectively. We also conducted an experiment in which glucose was added to block Cas13a protein expression. It was observed that the proportion of *S.* Typhimurium was not less than 50% in the majority of cases after glucose supplementation (Fig. S7). These data confirmed that the CRISPR-Cas13a system could reduce the proportion of *S.* Typhimurium in a microflora.

### cCKPs prevent *S*. Typhimurium colonization in the mouse gut.

**FIG 6:**
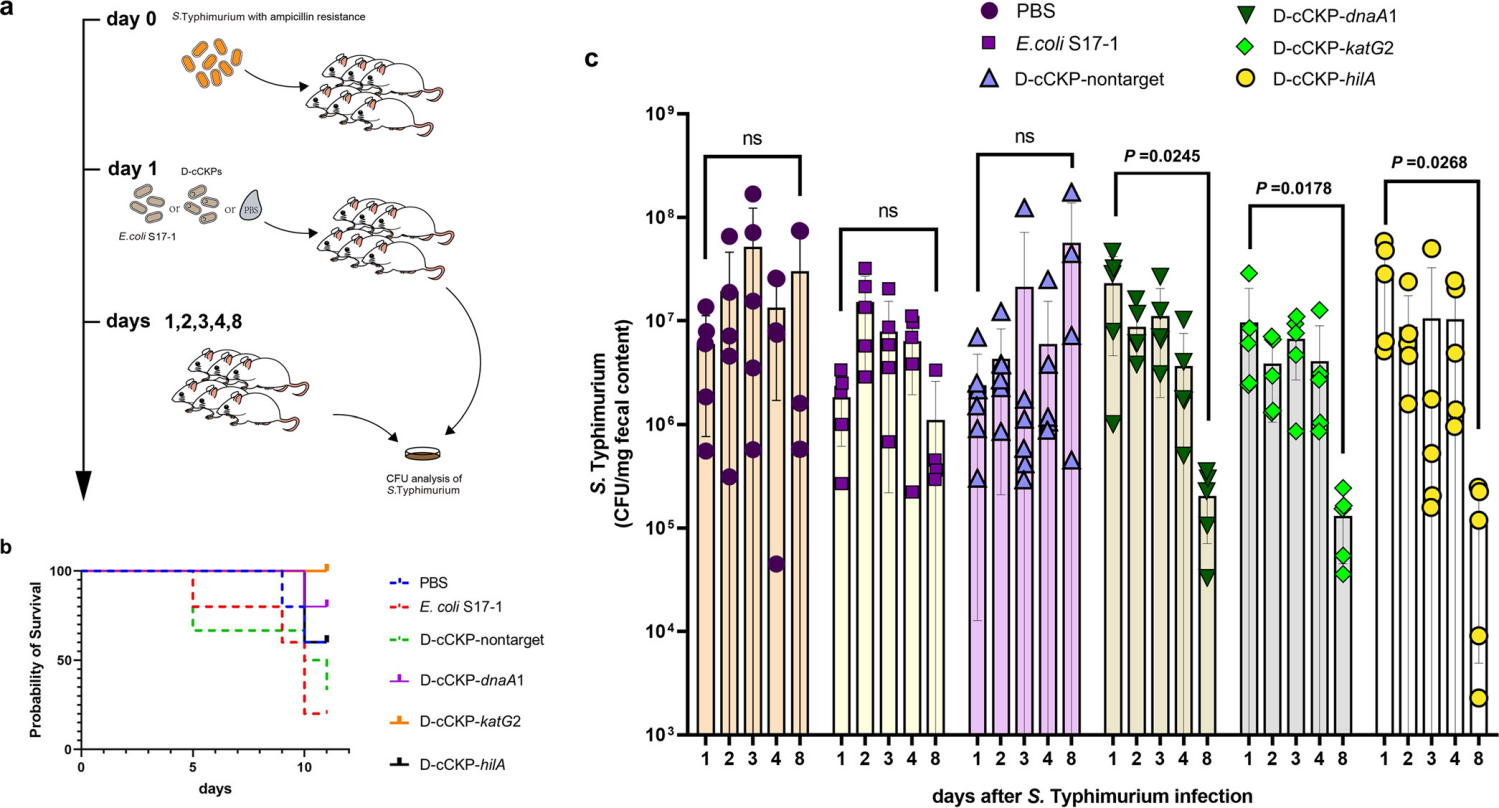
CRISPR-Cas 13a reduces *S.* Typhimurium colonization in a mouse model. (a) Diagrams showing the key steps of the mouse experiments. (b) Mouse survival rate after inoculation with donor strains. Vertical coordinate is the percentage of survival, and the horizontal coordinate is the number of days after *S*. Typhimurium infection. (c) *S.* Typhimurium CFU counts from fresh feces at the indicated time points from different groups inoculated with E. coli S17-1, d-cCKP-nontarget, d-cCKP-*dnaA*1, d-cCKP-*katG*2, or d-cCKP-*hilA*. Bars represent the standard deviation of data from 5 to 6 biological replicates, dots represent each duplicate data, and *P* values were determined by two-sided Student’s *t* test. Each group consisted of 5 to 6 mice, but some mice died during the experiment, leading to a decrease in some data points in some groups.

Given the efficacy of the CRISPR-Cas13a system in killing and inhibiting the growth of *S.* Typhimurium *in vitro*, we sought to determine whether it would affect *S.* Typhimurium infection in a mouse model *in vivo*. Mice were initially administered 2 mg/mL ampicillin in their drinking water for 3 days to render them susceptible to the colonization of ampicillin-resistant *S.* Typhimurium and then challenged with ~10^9^ CFU of *S.* Typhimurium on day 0. One day later, groups of mice were inoculated with ~10^9^ CFU of E. coli S17-1 without plasmid, four d-cCKP (donor with cCKPs) strains, or an equivalent volume of PBS. Mouse feces were collected on days 1, 2, 3, 4, and 8 postchallenge (Fig. 6a). We chose 62 colonies on ampicillin plates and used primers F-hilA and R-hilA for colony PCR to confirm that the colonies were *S.* Typhimurium. All the samples showed positive results (~1,700 bp), indicating that the colonies were *S.* Typhimurium. Meanwhile, we determined the conjugation frequency *in vivo*, and we found that the conjugation efficiency *in vivo* reached ~10% within 3 days and was subsequently approximately at a steady state (Fig. S8).

We observed a slight increase in the number of CFU of *S.* Typhimurium from feces at day 2 in groups inoculated with PBS, E. coli S17-1, and d-cCKPs-nontarget (Fig. 6c). In contrast, all three d-cCKPs targeting transcripts showed an approximately 3-fold decrease in the number of targeted bacteria at day 2 (Fig. 6c). On day 8 postchallenge, we observed no significant decline in the number of CFU of *S.* Typhimurium in the groups inoculated with PBS, E. coli S17-1, and d-cCKP-nontarget, while other d-cCKPs had a significant decrease in the number of *S.* Typhimurium. Specifically, the d-cCKP-*dnaA*1 group decreased ~115-fold (from 2.31 × 10^7^ to 2.04 × 10^5^, *P *= 0.0245), the d-cCKP-*katG*2 group decreased ~74-fold (from 9.64 × 10^6^ to 1.30 × 10^5^, *P = *0.0178), and the d-cCKP-*hilA* group decreased ~2.4 orders of magnitude (from 2.92 × 10^7^ to 1.21 × 10^5^, *P = *0.0268). The population density of *S.* Typhimurium did not decrease significantly on days 2 to 4, which we suspect may be related to the lower conjugation frequency at this time (Fig. S8). At the same time, cCKPs took a period of time to exhibit a bactericidal effect; this lag effect may also lead to the insignificant reduction of population density on days 2 to 4.

We also determined the survival rate of mice within 11 days after challenge with *S.* Typhimurium. The earliest deaths occurred in the groups inoculated with E. coli S17-1 and d-cCKP-nontarget on day 5 postchallenge, and 2 and 1 died, respectively. By day 11 postchallenge, the lowest survival rates occurred in the groups inoculated with E. coli S17-1 (20%) and d-cCKP-nontarget (30%). The survival rates were 60% for the groups inoculated with PBS and d-cCKP-*hilA*, 80% for the group inoculated with d-cCKP-*dnaA*1, and 100% for the group inoculated with d-cCKP-*katG*2 (Fig. 6b). At the same time, we observed that the mortality rate of mice in the PBS group was lower than that in the d-cCKP-nontarget and E. coli S17-1 groups, and we speculated that this might be due to the intestinal infection caused by E. coli after inoculation with the *S.* Typhimurium pathogen caused by further disturbance of the tract flora. Since d-cCKPs could kill *S.* Typhimurium and reduce its toxic effects, the disturbance of tract flora and toxic effects cancel each other out, resulting in an increased survival rate. Overall, these results showed that the CRISPR-Cas13a system targeting the endogenous transcriptome through a conjugation vehicle reduced the population density of pathogenic Salmonella in the mouse gut and increased the survival rate.

## DISCUSSION

We constructed a CRISPR-Cas13a system targeting endogenous transcripts to specifically kill *S.* Typhimurium by conjugative delivery. We demonstrated the efficacy of this system in the selective killing of Salmonella by using *in vitro* and *in vivo* experiments. The results prove the feasibility of the approach and provide information for future improvement. However, CRISPR-Cas13 also has some disadvantages. For example, it acts on the transcriptome and has promiscuous RNA cleavage activity, resulting in the inability to eliminate plasmids carrying drug resistance genes, which can be achieved by CRISPR-Cas9. Therefore, the concatenation of multiple CRISPR systems may be needed to further increase the bactericidal efficiency and will be examined in future studies. Additionally, due to the RNA recognition activity, the catalytically inactive Cas13 enzyme (dCas13) protein is also a potentially useful tool to achieve accurate expression inhibition of target RNA-programmable tracking of transcripts without random cleavage activity (40).

In this study, we selected three *S.* Typhimurium genes as targets, the *dnaA* gene for crRNAs *dnaA*1 and *dnaA*2, the *katG* gene for crRNAs *katG*1 and *katG*2, and the *hilA* gene. The gene *dnaA* is essential for the growth of bacteria, but the other two are not. However, based on the results of experiments *in vitro* and *in vivo*, we found that the bactericidal growth inhibition efficiencies for crRNAs showed similar resultant targeting of all three genes, which implied that the bactericidal effects caused by CRISPR-Cas13a were mainly due to random cleavage. In addition, the CRISPR-Cas13a system reduced the expression of *katG* and *hilA*, resulting in hydrogen peroxide detoxification defect or HilA regulatory network distortion, which might enhance bactericidal effect. As shown in Fig. 2c, although targeting the same gene, *dnaA*, crRNA *dnaA*1, and *dnaA*2 also had a large difference in elimination effect (one is ~29%, and the other is ~87%), This might indicate that the CRISPR-Cas13a system has differential activation for different target sequences. Under the conditions of elimination experiments *in vitro* (result shown in Fig. 2c), the lower activity of bacteria that just finished conjugation resulted in lower *dnaA* mRNA content (41), and the *dnaA*1 group had lower binding activity that mediated more colonies escaping the bactericidal effect because of the lack of enough active CRISPR-Cas13a systems. But in cell death assay experiments and growth inhibition experiments (results shown in Fig. 3 and 5, respectively), strains we used had higher activity (*A*_600_~0.5) and higher *dnaA* mRNA content. Although the binding efficiency of the *dnaA*1 group is low, a high content of target RNA mediated enough active CRISPR-Cas13a systems reaching the bactericidal threshold in more cells, so groups showed similar results. Therefore, designing the optimal crRNA sequence is also one of the problems we are facing. Wessels et al. conducted massively parallel screens targeting mRNAs and developed a computational model to choose optimal crRNAs for Cas13b (42). Design method optimization for Cas13a remains to be resolved. Coupled with the tools to optimize crRNA, the prospects of Cas13a as a bactericidal tool will be much better.

The method of conjugative transfer, although it can continuously transfer CKPs into flora, has a major disadvantage of low efficiency (approximately 5 × 10^−2^ as determined by *in vitro* experiments in this study). Various methods have been explored to enhance the conjugation efficiency. Hamilton et al. increased the efficiency of conjugation by promoting cell-to-cell contact using beads *in vitro* (23). Neil et al. increased the conjugation efficiency through accelerated laboratory evolution approaches, improving the conjugation efficiency by approximately 3 orders of magnitude (33). Yu et al. showed that nonnutritive sweeteners can increase the rate of conjugation, and the use of a specific coating to allow the release of nonnutritive sweeteners at the site of conjugation donor action is also a possible approach (43). In addition, *cis*-conjugative delivery (conjugative genes and antimicrobial genes are put in a single plasmid) is also a convenient, excellent method that is not required for modification in conjugative machines or donor strains. In this approach, nontarget strains that accepted the antimicrobial plasmid will act as donors, increasing the ratio of donors in flora. Hamilton et al. used *cis*-conjugation, reaching a maximum conjugative frequency of 1 × 10^−2^ by 24 h (23). These systems could be used in future efforts to optimize conjugation methods.

We admit that every CRISPR-based bactericidal method has potential problems for escape. In a previous study, the occurrence of rearrangements of plasmid or mutations in the CRISPR cassette and target sites might lead to bactericide failure (23, 44). In this study, we collected 25 “escaped” colonies from 5 target CKP groups in arabinose plates under the condition of elimination assay experiments. We cultured and sequenced them to assay the mutant sites in the target sequence or Cas13a cassette. Four of the colonies could not be cultured in LB broth with chloramphenicol, kanamycin, and ampicillin, indicating the loss of CKPs. Surprisingly, only one showed a frameshift mutation in the Cas13a cassette, and the rest of the 20 colonies exhibited no mutation in target sites and Cas13a system. We speculate that this phenomenon may cause by mutations at some other sites which reduce or neutralize the toxicity of Cas13a RNA-cutting activity. In brief, how to reduce the number of escapes is a critical problem for the CRISPR-based bactericidal method.

In addition, anti-CRISPR protein, redundant crRNA, plasmid incompatibility, and promoter activity are also potential hurdles for practical application. Because of the need for a donor strain to deliver the plasmid, the fitness cost induced by CRISPR cassette expression (as shown in Fig. 3b and Fig. 4b) may lead to accelerated plasmid loss, rearrangements, and selection of mutations that downregulate Cas13a expression in donor strains. To minimize the occurrence of the above situation, we can use species-specific promoters to control the expression of Cas13a, which cannot only reduce the metabolic burden caused by the constitutive expression CRISPR system but also further improve the specificity of the bactericidal system (45). Also, the stability of the plasmid in the donor strain is also important. Toxins, antitoxin systems (46, 47), and auxotrophic methods (48) can be used to increase the stability of the plasmid in the donor. Additionally, microbial therapies face some common problems, such as biosafety issues, which can be reduced by introducing temperature control, community density control, or induced suicide switching (49–51) in microorganisms. Self-targeted crRNAs may be used in conjunction with CRISPR as a suicide system and can avoid the increased workload due to the introduction of exogenous suicide systems. With continued technological improvement and optimization, pathogen-specific microbial therapies will become a reality.

## MATERIALS AND METHODS

### Bacterial strains and culture conditions.

E. coli *Trelief 5α F^−^*φ80(*lacZ*)ΔM15 Δ(*lacZYA-argF*)*U169 deoR endA1 recA1 hsdR17* (r_K_*^−^* m_k_*^+^*) *supE44λ thi-1 gyrA96 relA1 phoA* (Tsingke Biological Technology, Beijing, China) was used for plasmid construction and as a conjugative recipient (plasmid pUC19 with ampicillin resistance gene was transferred into this strain). *S.* Typhimurium with ampicillin and tetracycline resistance activation was used as a conjugative recipient (our lab collection). E. coli S17-1 RP4-2(km::Tn*7*,Tc::Mu-1), *pro-82* λ*pir recA1 endA1 thiE1 hsdR17 creC510* was used as a conjugative donor for transferring CKPs to Salmonella.

Bacteria were cultured at 37°C in Luria-Bertani (LB) media (10 g/L tryptone, 5 g/L yeast extract, and 10 g/L NaCl). When needed, other chemicals were added at the following concentrations: 100 μg/mL for ampicillin, 50 μg/mL for kanamycin, 34 μg/mL for chloramphenicol, and 2 g/L (0.2%) for arabinose and glucose.

### Plasmid construction.

The primer sequences used for plasmid construction are shown in Table S2 in the supplemental material. First, the fragment of the linear backbone containing the plasmid-origin pBBR1, mob gene, origin of transfer (*oriT*), and kanamycin resistance gene was amplified from pBBR1-MCS2 using primers F-pBBR1 and R-pBBR1. The LwaCas13a cassette with a crRNA cassette was amplified from pC011, a gift from Feng Zhang (Addgene; plasmid number 91903; http://n2t.net/addgene:91903; RRID Addgene_91903) (29), using primers F-Cas13a-crRNA and R-Cas13a-crRNA. The segment containing the *araC* gene and pBAD was amplified from E. coli S17-1 using primers F-araC and R-pBAD. These three fragments had 15- to 25-bp homologous overlaps and were assembled by a ClonExpress II one-step cloning kit (Vazyme Biotech Co., Ltd., Nanjing, China) to construct the plasmid pre-CKP, which was transformed into E. coli Trelief 5α (Tsingke Biological Technology). Then, we used HindIII (TaKaRa Bio, Dalian, China) to digest the purified pre-CKP and amplified the chloramphenicol resistance gene, *cmR,* from pC011 using primers F-CmR-insert and R-CmR-insert with a 15-bp overlapping homologous arm to the linear pre-CKP. Using ClonExpress, these fragments were assembled into a circular plasmid CKP-nontarget and transformed into E. coli Trelief 5α chemically competent cells. Other crRNAs were synthesized by the DNA synthesis method, and nontarget crRNA was removed by the AvrII and PacI double-digestion method from CKP-nontarget. The crRNAs, including *dnaA*1, *dnaA*2, *katG*1, *katG*2, and *hilA*, were individually inserted into the CKP backbone and replaced the nontarget crRNA. (Table S1).

To construct cCKPs, CKPs were used as a template, and primers F-J23104-RBS-Cas13a, R-RBS-Cas13a, and R-J23104-RBS-Cas13a were used to construct the constitutive promoters J23104 and RBS B0034 and amplify the LwaCas13a cassette, crRNA, and chloramphenicol resistance gene. The fragment containing the origin and replication protein genes of the pBBR1, *oriT*, and kanamycin resistance gene was amplified using the primers F-pBBR1-cckp and R-pBBR1-cckp. Two fragments had 15-bp overlapping homologous arms using ClonExpress to assemble these parts, which were transformed into E. coli Trelief 5α chemically competent cells to obtain cCKPs.

### Conjugation assay.

The recipient *S.* Typhimurium and donor d-CKP strains were cultured overnight in LB broth supplemented with the appropriate antibiotic. Overnight cultures were subcultured 1:100 into LB and incubated at 37°C to an *A*_600_ of 0.5. Then, the recipient *S.* Typhimurium was mixed 1:1 (500:500 μL) or 1:2 (333:667 μL) with the individual donor d-CKP strain. The mixtures were centrifuged at 5,000 × *g* for 5 min, LB broth was removed, and the cells were resuspended in 100 μL of 1× PBS. These mixtures were used for conjugation in different media. For filter media, the 0.5-μm filters were placed on LB agar plates, and the bacterial mixture was dropped on the filters. For agar or broth media, 100 μL of the mixture was directly plated onto nonselective LB plates or added to LB broth. Conjugation proceeded at 37°C for 20 h (LB broth medium was also at 180 rpm). After conjugation, 1 mL PBS was used to wash out cells from filters. Tenfold serial dilutions down to 1:10^6^ were made in LB, and a 5-μL volume of each dilution was spotted onto LB agar supplemented with 0.2% glucose and the antibiotics ampicillin, kanamycin, and chloramphenicol or with ampicillin alone to assay the conjugative frequency.

### *In vitro* elimination of *S.* Typhimurium by conjugation.

Filter conjugation was used as described above. After conjugation, 10-fold serial dilutions down to 1:10^6^ were made in LB, and a 5-μL volume of each dilution was spotted onto LB agar supplemented with 0.2% glucose or 0.2% l-arabinose and appropriate antibiotics to assay the elimination efficiency. Using the CFU numbers, the elimination efficiency was calculated as follows:

$$\text{elimination efficiency}=1-\frac{\text{CFUs of transconjugants with arabinose}}{\text{CFUs of transconjugants with glucose}}.$$

### Demonstration of cell death caused by CRISPR-Cas13a.

*S.* Typhimurium-carrying CKPs were cultured in LB broth with ampicillin, kanamycin, chloramphenicol, and 0.2% glucose overnight. The cells were diluted at a ratio of 1:100 in LB broth and cultured to an *A*_600_ of ~0.5 to obtain exponentially growing cells. Then, the mixture of 1,000 μL PBS supplemented with 0.2% arabinose or glucose and 10 μL bacterial solution was cultured at 37°C and 180 rpm; sampled at 0 h, 12 h, and 24 h; and diluted from 10^−4^ to 10^−8^. The inoculum was cultured in recovery LB medium containing 0.2% glucose, ampicillin, kanamycin, and chloramphenicol, and the CFU were enumerated. For spot-plating images, about 10 to 15 μL of sample diluent was plated at different dilution folds in recovery LB medium and cultivated at 37°C for 16 h. Photographs were taken for spot-plating images.

### Growth curve.

CKP-carrying *S.* Typhimurium strains were cultured in LB broth supplemented with 100 μg/mL ampicillin, 50 μg/mL kanamycin, 34 μg/mL chloramphenicol, and 0.2% glucose overnight (the CKP-free *S.* Typhimurium strain used for the control group was cultured in LB broth supplemented with 100 μg/mL ampicillin and 0.2% glucose overnight). The cells were diluted at a ratio of 1:10 in LB broth and cultured to an *A*_600_ of ~0.5 to obtain exponentially growing cells. Then, 30 mL of LB broth with 0.2% arabinose or glucose and the antibiotics ampicillin, kanamycin, and chloramphenicol and 300 μL of exponentially growing bacterial solution were added to the test bottles. The bottles were incubated in a microscreen growth curve tester (Gering, Tianjin, China) at 37°C and 400 rpm, and *A*_600_ measurements were performed every 10 min for 16 h.

### Flora exclusion experiment.

CKP-carrying E. coli Trelief 5α strains and CKP-carrying *S.* Typhimurium strains were cultured in LB broth supplemented with 100 μg/mL ampicillin, 50 μg/mL kanamycin, 34 μg/mL chloramphenicol, and 0.2% glucose overnight. Overnight strains were diluted at a ratio of 1:100 in LB broth and cultured to an *A*_600_ of ~0.5. E. coli and *S.* Typhimurium were mixed at a ratio of 1:1. The mixture was cultured in LB broth with 0.2% arabinose or glucose and the antibiotics ampicillin, kanamycin, and chloramphenicol at 37°C and 220 rpm and sampled at 0 h, 6 h, and 12 h. The collections were serially diluted 10-fold and cultured in recovery LB plates (containing 0.2% glucose) supplemented with ampicillin or tetracycline to calculate the community density of the total flora or *S.* Typhimurium. The number of CFU was enumerated. The proportion of *S.* Typhimurium in the community was calculated using the following formula:

$$\text{proportion of }S.\text{ Typhimurium}=\frac{\text{CFU in plates with tetracycline}}{\text{CFU in plates with ampicillin}}.$$

### Mouse model for Salmonella infection.

All animal protocols were approved by the Animal Care and Use Committee of the Model Animal Research Center, College of Life Sciences, Sichuan University. Six- to 8-week-old ICR/KM mice were purchased from a local company (Dossy Experimental Animals Co., Ltd., Chengdu, China). Then, they were randomly assigned to 6 groups with 5 or 6 mice in each group. Mice were housed in cages with *ad libitum* access to water and commercial feed. After 3 days of acclimation, mice were given 2 mg/mL ampicillin in their drinking water for 3 days to render them susceptible to the colonization of ampicillin-resistant *S.* Typhimurium and then challenged with ~10^9^ CFU of *S.* Typhimurium. One day later, the mice were orally inoculated with 1 × 10^9^ CFU of E. coli S17-1 or d-cCKP-*dnaA*1, d-cCKP-*katG*2, d-cCKP-*hilA*, or d-cCKP-nontarget or an equal volume of PBS.

Mouse feces were collected on days 1, 2, 3, 4, and 8 after inoculation with *S.* Typhimurium using a 1.5-mL centrifuge tube. The feces were weighed, and 500 mL PBS solution and 1 to 3 glass beads (Sangon Biotech, Shanghai, China) were added to crush the feces under a vortex mixer (Sangon Biotech, Shanghai, China). The fecal solution was serially diluted from 10^−3^ to 10^−7^, plated on LB agar plates supplemented with ampicillin, and incubated at 37°C for 16 h. The number of CFU was enumerated.
