# Supplementary material for: Pathogen-Specific Bactericidal Method Mediated by Conjugative Delivery of CRISPR-Cas13a Targeting Bacterial Endogenous Transcripts
Source: Microbiol Spectr. 2022 Aug 11;10(4):e01300-22. doi: 10.1128/spectrum.01300-22 (PMC9430969; doi:10.1128/spectrum.01300-22)
Supplement: Supplemental file 1 — Supplemental material. Download spectrum.01300-22-s0001.pdf, PDF file, 1.4 MB [file spectrum.01300-22-s0001.pdf]

SUPPLEMENTARY FIGURES AND FIGURE LEGENDS

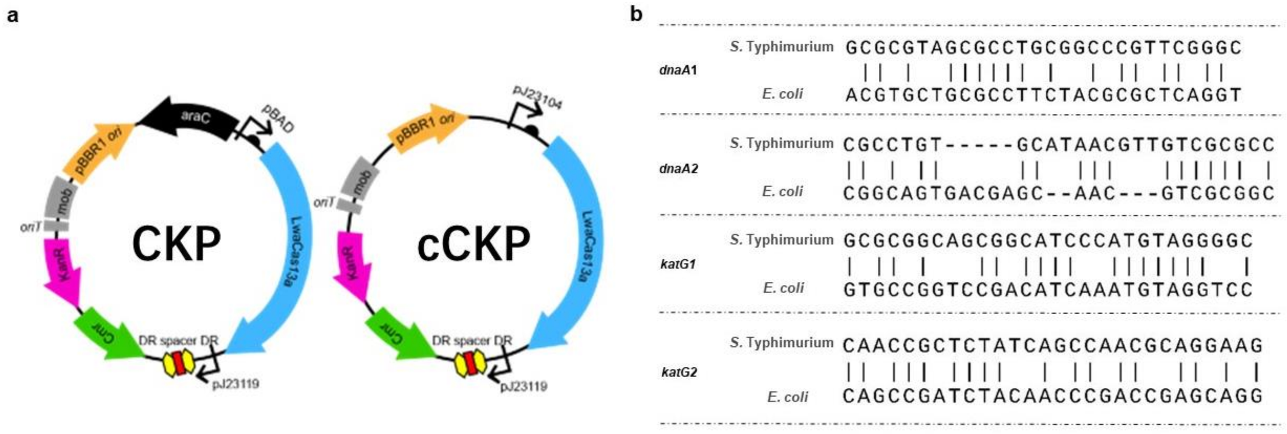

Figure S1. Schematic diagrams of plasmids CKP and cCKP and crRNA target sequence alignment. (a) All CKP and cCKP plasmids contain a pBBR1 broad host origin, a mob gene (encode relaxase), an origin of transfer (oriT), kanamycin and chloramphenicol resistance genes, a CRISPR RNA expression cassette, and a LwaCas13a gene with ribosome binding sites (RBS). For the CKPs used in the *in vitro* experiments (left panel), LwaCas13a was controlled by the promoter pBAD. For the *in vivo* experiments, we replaced pBAD with the constitutive promoter pJ23104 to control LwaCas13a, constituting cCKPs (right panel). (b) crRNA target sequence alignment between *E.coli* and *S.* Typhimurium.

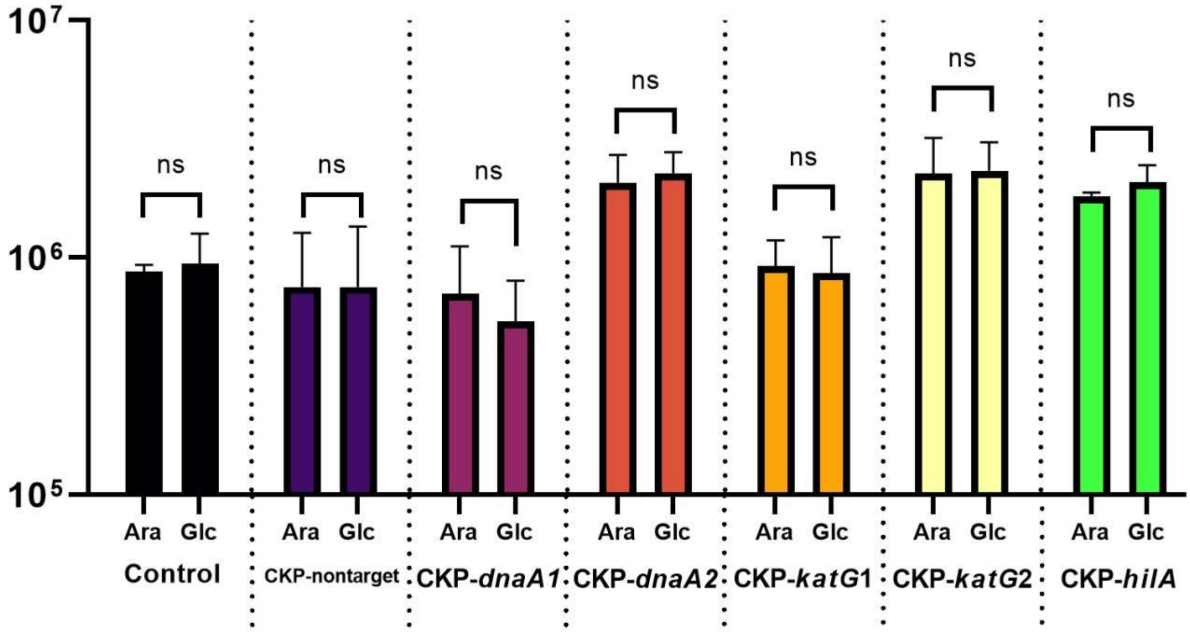

Figure S2. CKPs do not mediate elimination to *E. coli*. We repeated the experiment shown in Fig. 2c using *E. coli* as the recipient. Bars represent the standard deviation of data from 3 biological replicates, dots represent each duplicate data, and *P* values were determined by ratio paired Student's t test (ns, $P > 0.05$)

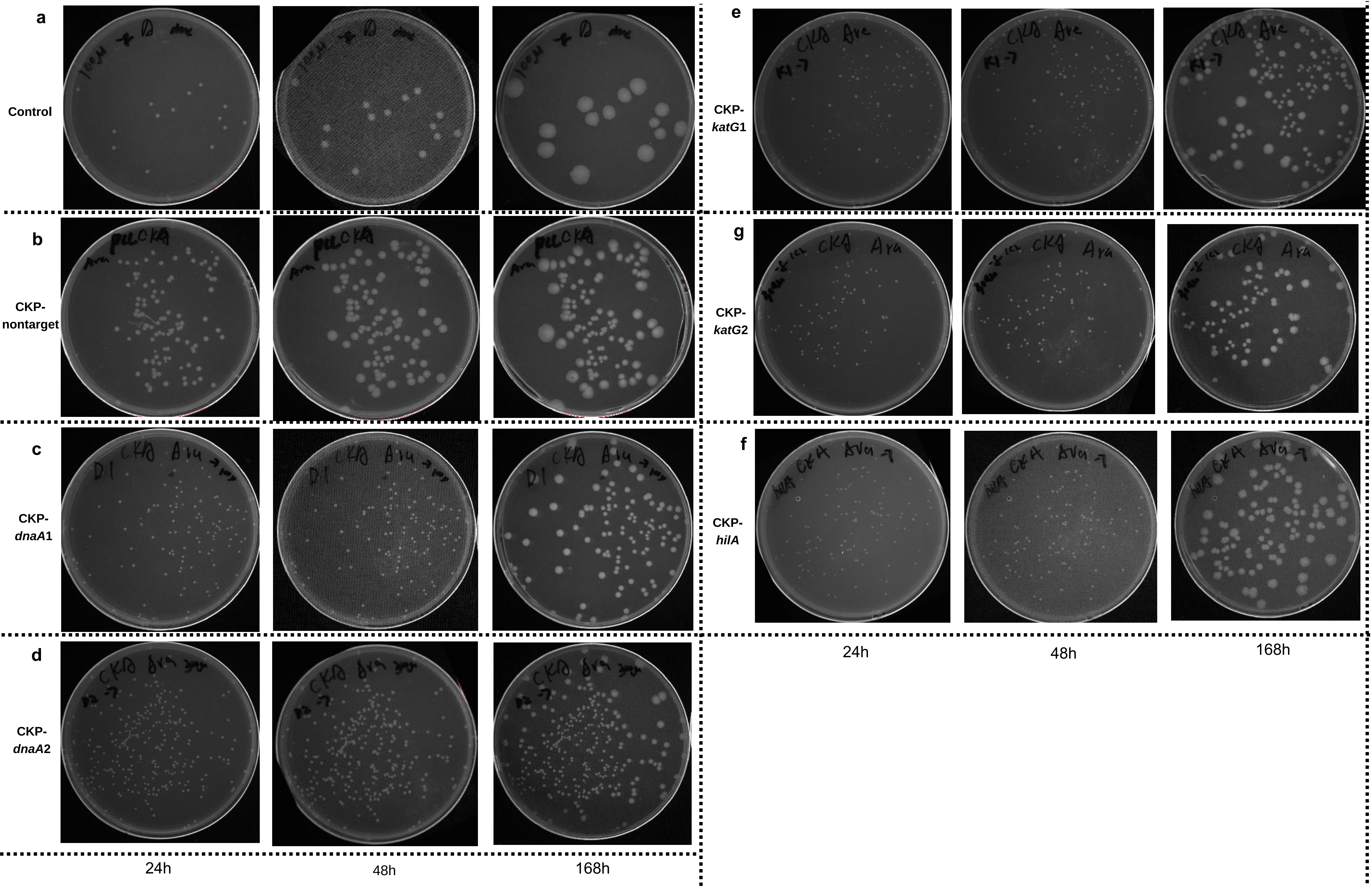
a
Control
b
CKP-nontarget
c
CKP-dnaA1
d
CKP-dnaA2
24h
48h
168h
e
CKP-katG1
g
CKP-katG2
f
CKP-hilA
24h
48h
168h

Figure S3. Images of colonies in arabinose LB plates. Following the method in fig. 2c cultured the arabinose-added plate for 168h and colony growth conditions were observed.

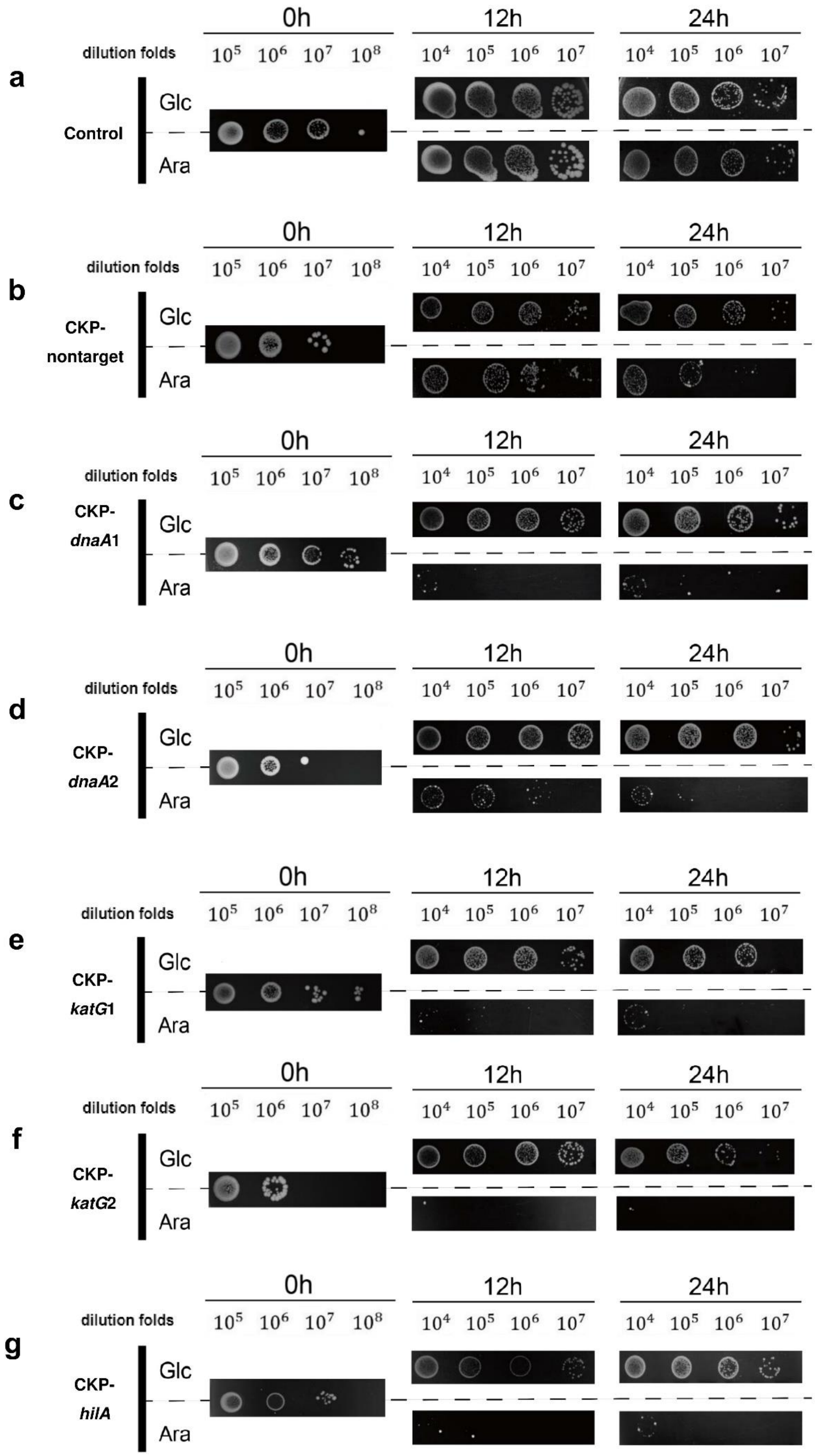
a
0h
12h
24h
dilution folds
10^5 10^6 10^7 10^8
10^4 10^5 10^6 10^7
10^4 10^5 10^6 10^7
Control
Glc
Ara
b
0h
12h
24h
dilution folds
10^5 10^6 10^7 10^8
10^4 10^5 10^6 10^7
10^4 10^5 10^6 10^7
CKP-nontarget
Glc
Ara
c
0h
12h
24h
dilution folds
10^5 10^6 10^7 10^8
10^4 10^5 10^6 10^7
10^4 10^5 10^6 10^7
CKP-*dnaA*1
Glc
Ara
d
0h
12h
24h
dilution folds
10^5 10^6 10^7 10^8
10^4 10^5 10^6 10^7
10^4 10^5 10^6 10^7
CKP-*dnaA*2
Glc
Ara
e
0h
12h
24h
dilution folds
10^5 10^6 10^7 10^8
10^4 10^5 10^6 10^7
10^4 10^5 10^6 10^7
CKP-*katG*1
Glc
Ara
f
0h
12h
24h
dilution folds
10^5 10^6 10^7 10^8
10^4 10^5 10^6 10^7
10^4 10^5 10^6 10^7
CKP-*katG*2
Glc
Ara
g
0h
12h
24h
dilution folds
10^5 10^6 10^7 10^8
10^4 10^5 10^6 10^7
10^4 10^5 10^6 10^7
CKP-*hilA*
Glc
Ara

Figure S4. Spot plating experiments.

Spot plating images show the *S.* Typhimurium in different dilution folds.

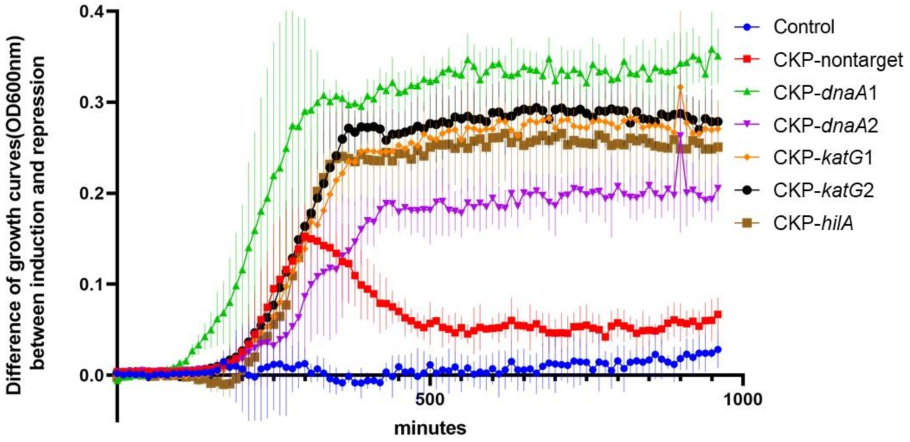

Figure S5. A_{600} differences over time between the arabinose-added culture and the glucose-added culture for each group. Data are based on Fig. **4a-4 g**.

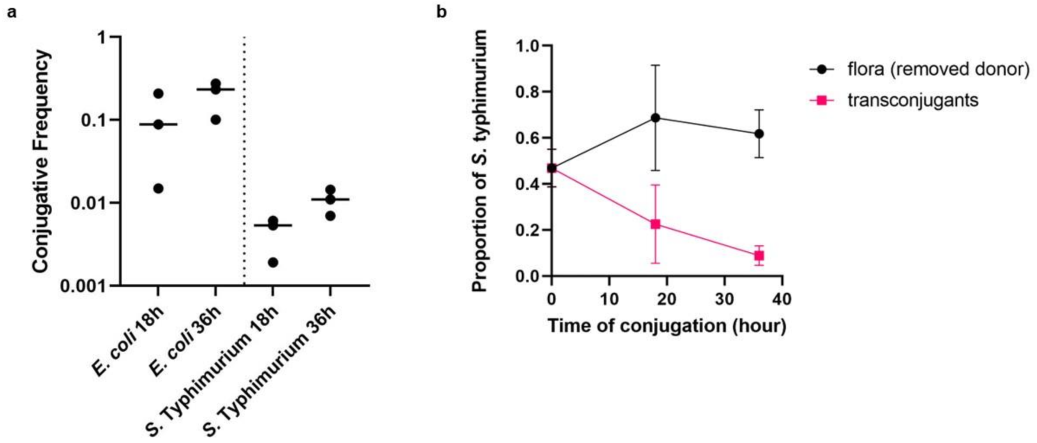

Figure S6. Conjugative frequency in flora and proportion of *S.* Typhimurium during the conjugation. (a) conjugative frequency in *E. coli*- *S.*

Typhimurium mixed flora (b) proportion of *S.* Typhimurium during the conjuation. Pink represents the proportion of *S.* Typhimurium in the flora of transconjugants and black represents the proportion of *S.* Typhimurium in the whole flora.

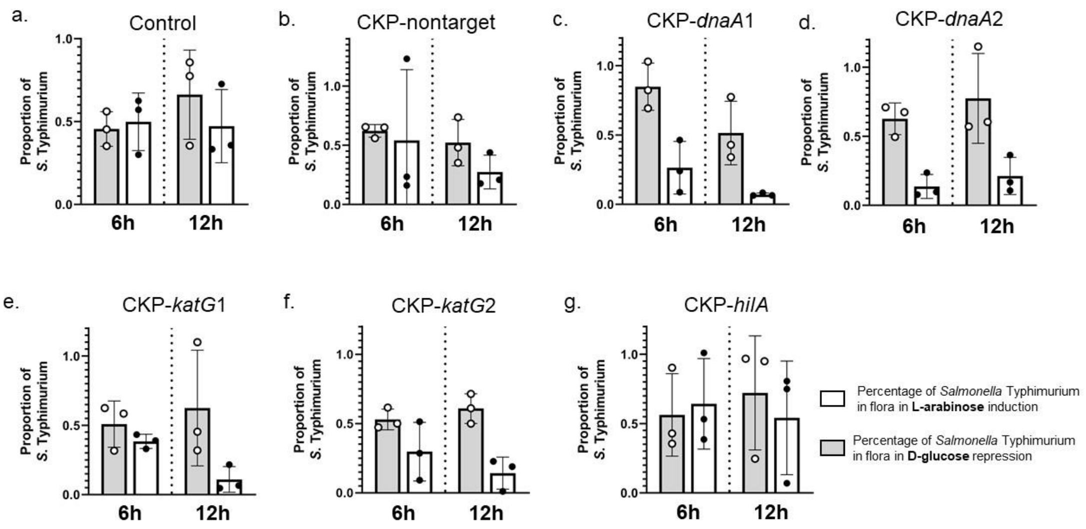

Figure S7. Proportion of *S.* Typhimurium in the mixedcommunity. The *S.* Typhimurium counts at the indicated time points between cultures supplemented with arabinose or glucose for each treatment group (labeled on top of each panel). Bars represent the standard deviation of data from 3 biological replicates, and dots represent each duplicate data point.

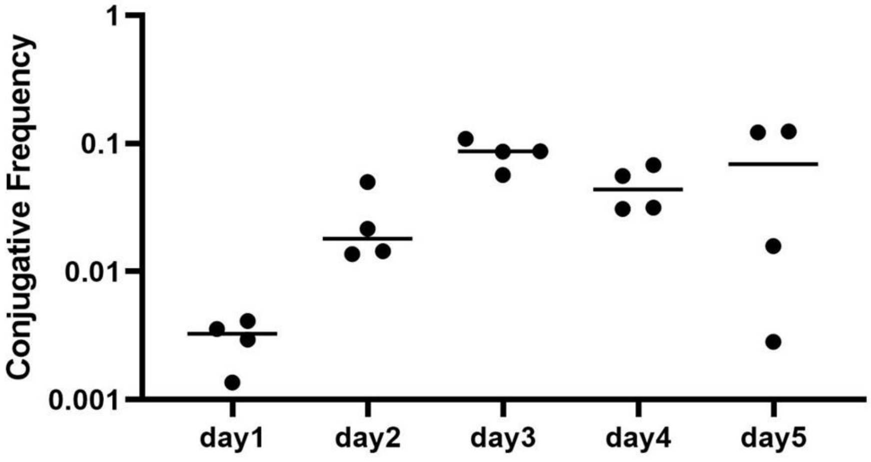

Figure S8.Conjugtive frequency in mice gut.

Day represents the time of the conjugation (Considered the Inoculate with d-cCKP-nontarget as day 0) and dots represent each duplicate data point.

SUPPLEMENTARY TABLES

Table. S1 crRNA sequences

*dnaA*1	gcccgaacgggccgcaggcgctacgcgc
*dnaA*2	gcaggcgcgacaacgttatgcacaggcg
*katG*1	gcccctacatgggatgccgctgccgcgc
*katG*2	cttcctgcgttggctgatagagcggttg
hilA	tgcatacttaataatattggattatcct
nontarget	agattgctgttctaccaagtaatccata

Table. S2 Primers used in this study

name	Sequence
F-araC	tttaagggcaccaataactgTTATGACAACTTGACGGCTACATCA
R-pBAD	gacctcctATGGAGAAACAGTAGAGAGTTGCGA
F-Cas13a-crRNA	gcttcggaatcgttttccggCGGTGCCTGACTGCGTTAGC
R-Cas13a-crRNA	ctgtttctccatAGGAGGTCTTATCCATGAAAGTGAC
F-pBBR1	aggcagacaaggtatagggcg
R-pBBR1	ccggaaaacgattccgaagcccaa

F-CmR-insert	taaactaccgcattaaagcttTGATCGGCACGTAAGAGGTTC
R-CmR-insert	gaaggggactaaaacaagcttCCTTAATTAAGGTTACGCCCCGCCCTGCCA
F-pBBR1-cckp	actgagctagctgtcaaaggcagacaaggtatagggcg
R-pBBR1-cckp	CGCAGTCAGGCACCGccggaaaacgattccgaagc
F-J23104-RBS-Cas13a	CGGTGCCTGACTGCGTTAGCaatttaactgtga
R-RBS-Cas13a	GTGCTAGCTACTAGAGAAAGAGGAGAAAATCTAGATATGAAAGTGACCAAGGTCGA
R-J23104-RBS-Cas13a	ttgacagctagctcagtcctaggtattgtgctagctactagagaaagagg
F-hilA (for colony pcr)	tcaccctgtaagagaatacactattatc
R-hilA (for colony pcr)	aatgcatatctcctctctcagatt

SUPPLEMENTARY METHODS

Conjugative frequency assay in flora and proportion of *S.* Typhimurium during the conjugation.

E. coli Trelief5α with pUC19 (with ampicillin resistance) and *S.* Typhimurium (with ampicillin and tetracyclin resistance) were cultured in LB broth supplemented with 100 µg/mL ampicillin overnight. D-CKP-nontarget was cultured in LB broth supplemented with 50 µg/mL kanamycin, 34 µg/mL chloramphenicol and 0.2% glucose overnight. Strains were diluted at a ratio of 1:100 in LB broth and cultured to A600 ~ 0.5. *E. coli Trelief5α* and *S.* Typhimurium were mixed at a ratio of 1:1 to 500 µL totally. The mixture was mixed with 500 µL D-CKP-nontarget and centrifuged at 5000 × g for 5 min, LB broth was removed, and the cells were resuspended in 100 µL of 1x PBS. The 0.5 µm filters were placed on LB agar plates, and the bacterial solution was dropped on the filters for 18 hours or 36 hours. Using 1 mL PBS to wash out cells on filters. The collections were serially diluted 10-fold and cultured in LB plates (containing 0.2% glucose) supplemented with different combination of antibiotics. The number of CFUs was enumerated. Datas were calculated using the following formulas. (Amp means ampicillin, Chl means chloramphenicol, Kan means kanamycin, Tet means tetracyclin)

$$\textbf{Conjugative frequency of } \boldsymbol{S}\textbf{. Typhimurium} = \frac{\text{CFUs in plates with Tet, Kan and Chl}}{\text{CFUs in plates with Tet}}$$

$$\textbf{Conjugative frequency of } \boldsymbol{E. coli} = \frac{\text{CFUs in plates with Amp, Cm and Kan} - \text{CFUs in plates with Tet, Kan and Chl}}{\text{CFUs in plates with Amp} - \text{CFUs in plates with Tet}}$$

$$\textbf{Proportion of } \boldsymbol{S}\textbf{. Typhimurium in transconjuagnts flora} = \frac{\text{CFUs in plates with Tet, Kan and Chl}}{\text{CFUs in plates with Amp, Kan and Chl}}$$

$$\textbf{Proportion of } \boldsymbol{S}\textbf{. Typhimurium in whole flora} = \frac{\text{CFUs in plates with Tet}}{\text{CFUs in plates with Amp}}$$

Conjugtion assay in mice gut

All animal protocols were approved by the Animal Care and Use Committee of the Model Animal Research Center, College of Life Sciences, Sichuan University. Six- to eight-week-old ICR/KM mice were purchased from a local company (Dossy Experimental Animals Co., Ltd., Chengdu, China). Four mice were housed in one cage with ad libitum access to water and commercial feed. After three days of acclimation, mice were given 2 mg/ml ampicillin in their drinking water for 3 days to render them susceptible to the colonization of ampicillin-resistant *S.* Typhimurium and then challenged with ~ 10^9 CFU of *S.* Typhimurium. One day later, the mice were orally inoculated with $1\text{x}10^9$ CFU D-cCKP-nontarget, considered the this day as day 0, which is different from the time calculation way in Fig. 6. Mouse feces were collected on days 1, 2, 3, 4, 5 after inoculation of D-cCKP-nontarget with a 1.5 mL centrifuge tube. The feces were weighed, and 500 mL PBS solution and 1-3 glass beads (Sangon Biotech, Shanghai, China) were added to crush the feces under a vortex mixer (Sangon Biotech, Shanghai, China). The fecal solution was serially diluted from 10^{-3} to 10^{-7}, plated on LB agar plates supplemented with ampicillin or with ampicillin, kanamycin, chloramphenicol and incubated at 37 °C for 16 h. The number of CFUs was enumerated. The conjugative frequency were calculated using the following formula.

$$\textbf{Conjugative frequency} = \frac{\text{CFUs in plates with ampicillin, kanamycin, chloramphenicol}}{\text{CFUs in plates with ampicillin}}$$
